# Spectrum-Effect Relationship between HPLC Fingerprints and Antioxidant Activity of Yangyin Tongnao Prescription

**DOI:** 10.1155/2021/6650366

**Published:** 2021-06-21

**Authors:** Li Yu, Yangyang Zhang, Xixi Zhao, Yu He, Haofang Wan, Haitong Wan, Jiehong Yang

**Affiliations:** ^1^School of Life Sciences, Zhejiang Chinese Medical University, Hangzhou, Zhejiang 310053, China; ^2^School of Basic Medical Sciences, Zhejiang Chinese Medical University, Hangzhou, Zhejiang 310053, China; ^3^School of Pharmaceutical Sciences, Zhejiang Chinese Medical University, Hangzhou, Zhejiang 310053, China; ^4^Academy of Chinese Medical Sciences, Zhejiang Chinese Medical University, Hangzhou, Zhejiang 310053, China

## Abstract

Yangyin Tongnao (YYTN) prescription is used as a traditional Chinese herbal formula, and it has antioxidant activity that mainly contributes in the treatment of cardiovascular and cerebrovascular diseases. However, the compounds related to its antioxidant activity are still unknown. In the present study, the fingerprints of YYTN extracts under different extraction conditions were obtained by high performance liquid chromatography (HPLC) to identify the common peaks to all the samples processed. A 2,2-diphenyl-1-picrylhydrazyl (DPPH) radical scavenging activity assay and ferric reducing antioxidant power (FRAP) assay were carried out to evaluate the antioxidant activity of the extracts. Spectrum-effect relationship between HPLC fingerprints and antioxidant activity of YYTN was assessed by Pearson product-moment correlation coefficient (PPMCC) and multiple linear regression analysis (MLRA). The results showed that peaks 5, 6, 13, 15, and 24 of the fingerprints were closely connected to antioxidant activity. Five peaks were identified: vanillic acid (*P5*), puerarin (*P7*), ferulic acid (*P13*), daidzein (*P21*), and formononetin (*P23*). Our study successfully established the spectrum-effect relationship between HPLC fingerprints and antioxidant activity of YYTN, which provided a general method for establishing quality standards with a combination of chromatography and antioxidant activity.

## 1. Introduction

Yangyin Tongnao prescription is a traditional Chinese herbal formula composed of six kinds of Chinese herbal medicines, *Radix Rehmanniae*, *Radix Astragali*, *Puerariae Lobatae Radix*, *Ligustici Chuanxiong Rhizoma*, *Dendrobii Caulis*, and *Hirudo.* However, four kinds of these Chinese herbal medicines except *Dendrobii Caulis* and *Hirudo* have been studied as a simplified formula in a lot of research [1, 2]. The proportions of herbal composition of Yangying Tongnao (YYTN) simplified prescription are shown in Table 1 [3]. The efficacy of YYTN has been evaluated by clinical trials and animal experiments. According to the Chinese medicine, it has the effects of nourishing yin and replenishing qi (deficiency of yin or qi is a kind of syndrome; deficiency of yin usually appears hot and bothered, and deficiency of qi has a feeling of weariness). In the Western medicine, it could promote blood circulation, especially in the treatment of cardiovascular and cerebrovascular diseases [4]. Previous researches have proved that YYTN had the functions of inhibiting neuronal apoptosis, improving haematological disorders [5], and is related with antioxidant and anti-inflammatory properties [6]. As a traditional Chinese herbal formula, YYTN can also improve the neurological deficits after focal cerebral ischemic infarction in rats [3]. At the same time, it was found that YYTN has protective effect on oxidative stress-induced cerebral ischemia injury *in vivo* through animal experiments [7]. However, there does not seem to be any report about the relationship between active ingredients and pharmaceutical efficacy of YYTN.

Antioxidants play a crucial role in preventing various serious diseases caused by oxidative stress damage. At present, several methods have been applied to evaluate the antioxidant activity, including assays for ferric reducing antioxidant power (FRAP) [8–10], 2, 2-diphenyl-1-picrylhydrazyl (DPPH) radical scavenging [11–13], and hydroxyl radical scavenging [14, 15]. Therefore, a growing number of researchers are paying attention to the search for natural compounds from plants or vegetal drugs with antioxidant properties [16–18]. However, the compounds of YYTN responsible for its antioxidant activity are unclear.

Nowadays, spectrum-effect is used to study the relationship between components and efficacy of traditional Chinese medicines. For example, the main vasorelaxant components of rosemary have been found by using the chemical spectrum-effect relationship [19]. HPLC fingerprint and bioactivity verification were used to investigate the potential correlation between the chemical components and pharmaceutical effects [20, 21]. It is a representative method to find the main active components in complex traditional Chinese herbal formulas based on the study of spectrum-activity relationship.

There are three major categories of data processing methods for spectrum-effect relationships, such as correlation analysis, cluster analysis, and regression analysis. Correlation analysis is a statistical method for studying the degree of closeness between variables [22], including grey relational analysis (GRA) and Pearson product-moment correlation coefficient (PPMCC). Cluster analysis is one of the important techniques in data mining and exploratory data analysis [23], including hierarchical clustering analysis (HCA) and non-hierarchical clustering analysis (N-HCA). Regression analysis refers to a statistical analysis method to determine the quantitative relationship between two or more variables [24], including multiple linear regression analysis (MLRA) and partial least squares (PLS). Therefore, GRA, PPMCC, PLS, and MLRA are applied to study the spectrum-effect relationship in the correlation analysis between the components and the efficacy of Chinese formulas [25–29].

In our study, HPLC fingerprints were combined with the data of the 2,2-diphenyl-1-picrylhydrazyl (DPPH) radical scavenging and ferric reducing antioxidant power (FRAP) assays to study the spectrum-effect relationship of YYTN using PPMC and MLRA. It lays the foundation for the pharmacokinetics substantial basis of YYTN.

## 2. Materials and Methods

### 2.1. Materials and Reagents

*Radix Rehmanniae*, *Radix Astragali*, *Puerariae Lobatae Radix*, and *Chuanxiong Rhizoma* were purchased from Traditional Chinese Medicine Pieces Co., Ltd. of Zhejiang Chinese Medical University (Hangzhou, China) and identified by Professor Shengwu Huang, Zhejiang Chinese Medical University (Hangzhou, China). HPLC-grade methanol and acetonitrile were obtained from Tedia Company, Inc. (Fairfield, OH, USA). Water was purified with a Millipore purification system (Millipore Co., Ltd, Billerica, MA, USA). DPPH was purchased from Shanghai Biochemical Co., Ltd. (Shanghai, China). FRAP kit was purchased from Nanjing Institute of Bioengineering (Nanjing, China). The reference standards of vanillic acid (≥99.0%), puerarin (≥98.0%), ferulic acid (≥98.0%), daidzein (≥99.0%), and formononetin (≥98.0%) were purchased from Shanghai Yuanye Co., Ltd. (Shanghai, China).

### 2.2. HPLC Fingerprints

#### 2.2.1. Chromatographic Conditions

HPLC analysis was performed using a Waters 2695 HPLC equipped with 2498 UV detector. The chromatographic separation was performed on a Hypersil BDS C18 column (4.6 mm × 300 mm, 5 *μ*m) maintained at 25°C. The mobile phase consisted of acetonitrile (*A*) and 0.5% formic acid water (B) with the following optimized gradient elution: 0 min: 2% A; 10 min: 10% A; 29 min: 12% A; 35 min: 15% A; 50 min: 25% A; 65 min: 60% A; 70 min: 90% A. The flow rate was 1.0 mL/min with an injection volume of 10 *μ*L, and the detection wavelength was set at 280 nm.

#### 2.2.2. Preparation of Standard Solutions

The mixed reference standard solution was prepared by adding 3 mg of vanillic acid, 36 mg of puerarin, 2.4 mg of ferulic acid, 6 mg of daidzein, and 1.8 mg of formononetin in a 10 mL volumetric flask and dissolved in 50% methanol to obtain final concentrations of 0.3, 3.6, 0.24, 0.6, and 0.18 mg/mL, respectively. Then the solution was filtered through a 0.22 *µ*m microporous membrane, and 50% methanol was added to final concentrations of 0.15, 0.075, 0.05, 0.0375, and 0.03 mg/mL of vanillic acid; 1.8, 0.9, 0.6, 0.45, and 0.36 mg/mL of puerarin; 0.12, 0.06, 0.04, 0.03, and 0.024 mg/mL of ferulic acid; 0.3, 0.15, 0.1, 0.075, and 0.06 mg/mL of daidzein; 0.09, 0.045, 0.03, 0.0225, and 0.018 mg/mL of formononetin for HPLC analysis.

#### 2.2.3. Preparation of Sample Solutions

The four herbs were crushed into crude grains. Accurately weighed 1.00 g of *Radix Rehmanniae*, 1.00 g of *Radix Astragali*, 1.20 g of *Puerariae Lobatae Radix*, and 0.67 g of *Chuanxiong Rhizoma* crude grains were mixed, and aqueous extracts were prepared using ultrasound-assisted extraction according to Tables 2 and 3. All the solutions were filtered through a 0.22 *µ*m microporous membrane and stored at 4°C until analysis.

#### 2.2.4. Validation of Methodology

Method precision was evaluated by five replicate injections of one sample, while repeatability was evaluated by five samples prepared independently from the same initial conditions. For the stability study, sample solutions were stored at room temperature and analyzed at 0, 2, 4, 8, 12, and 24 h, respectively.

#### 2.2.5. Peak Identification and Evaluation of Fingerprints

The reference standard solutions were injected into the HPLC system, and the chromatographic peaks in the sample solutions were identified according to the retention time of the reference substances.

Each sample solution and each standard solution was analyzed with the HPLC optimized condition to obtain the HPLC fingerprints. The fingerprints were matched automatically by the Similarity Evaluation System for Chromatographic Fingerprint of Traditional Chinese Medicine [30] (Version 2012A). Then the reference fingerprint was formed by the system using the median method from the comparison of nine samples, and the similarity values between the reference and nine samples were calculated using this software.

### 2.3. Antioxidant Activity Determination

#### 2.3.1. DPPH Assay

DPPH radical scavenging activity was determined according to Zhou et al. [31] with slight modifications. Briefly, 100 *µ*L of 0.4 mM DPPH anhydrous ethanol solution and 100 *µ*L of each sample solution at various concentrations were added to the 96 microplate. After incubation for 30 min at room temperature in the dark, the absorbance of the mixture was measured at 517 nm (SpectraMax Plus384 Absorbance Microplate Reader, Molecular Devices, USA). DPPH radical scavenging activity was calculated according to the following equation:

(1) $$\begin{array}{c} \text{scavenging percentage }\left(\text{\%}\right)=\left(1-\frac{A_{i}-A_{j}}{A_{0}}\right)\times 100, \end{array}$$

where *A*_i_ was the absorbance of sample + DPPH; *A*_j_ was the absorbance of sample + anhydrous ethanol; *A*_0_ was the absorbance of anhydrous ethanol + DPPH.

#### 2.3.2. FRAP Assay

The total antioxidant activity was evaluated by FRAP assay [32]. The FRAP solution was prepared by mixing 1.50 mL of 300 mmol/L acetate buffer (pH = 3.6) with 0.15 mL of 10 mmol/L TPTZ solution in 40 mmol/L HCl and 0.15 mL of 20 mmol/L FeCl_3_ solution, incubated for 30 min at 37°C in the dark. A fresh 100 mM FeSO_4_ solution was prepared from FeSO_4_.7H_2_O standard material. Then 5 *μ*L of the sample and 180 *μ*L of FRAP solution were added to the 96 microplate and allowed to react in the dark for 6 min at room temperature, and the absorbance was measured at 593 nm (SpectraMax Plus384 Absorbance Microplate Reader, Molecular Devices, USA). Aqueous standard solutions of 0.15, 0.3, 0.6, 0.9, 1.2, and 1.5 mmol/L of FeSO_4_ were used to generate a calibration curve. The final results were expressed as the equivalent concentration of the FeSO_4_ standard solution.

### 2.4. Spectrum-Effect Relationship

#### 2.4.1. Pearson Product-Moment Correlation Coefficient

The PPMCC is a statistical method to study the degree of closeness and direction of change between the two variables [33]. In this study, PPMCC was used to calculate the relationship between the peak area values of twenty-four common peaks to all the samples (*P1* to *P24*) in HPLC fingerprints and the antioxidant activities.

#### 2.4.2. Multiple Linear Regression Analysis

To eliminate the problem of multicollinearity that may exist among multiple independent variables, this study used the stepwise regression module in multiple linear regression in SPSS statistical software [34] to analyze the peaks of HPLC fingerprints, IC_50_ values of DPPH radical scavenging percentage, and the ferric reducing antioxidant power, respectively. Therefore, characteristic peaks with significant correlation among multiple independent variables were selected.

### 2.5. Statistical Analysis

The IC_50_ was calculated using the following URL: https://www.aatbio.com/tools/ic50-calculator. MLRA and PPMCC were performed by SPSS 25.0 software.

## 3. Results

### 3.1. Results of HPLC Fingerprints

#### 3.1.1. Method Validation

Method validation for HPLC fingerprint results showed that precision of the same sample solution was within the range of 0.19–1.37% for relative retention time and 0.75–2.67% for the average peak area of the common peaks to all the samples. Repeatability of the experiment appeared within the range of 0.15–1.04% for the relative retention time and 0.57–2.48% for relative peak area of common peaks. Sample stability was 0.11–1.32% for relative retention time and 0.69–2.74% for relative peak area of the common peaks to all the samples. All results indicated that the method of HPLC analysis was valid and satisfactory.

#### 3.1.2. HPLC Fingerprints

**Figure 1:**
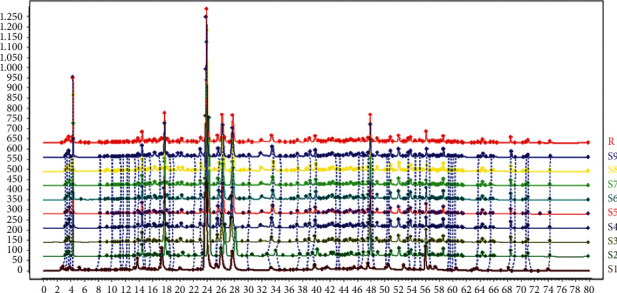
HPLC fingerprints of YYTN and their reference (the detection wavelength was set at 280 nm, and the time is expressed in minutes).

**Figure 2:**
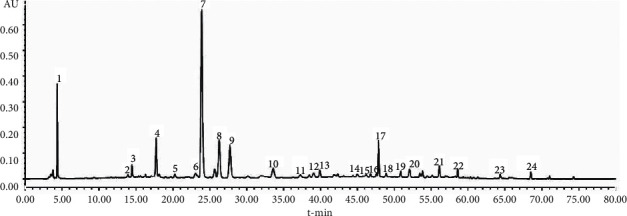
The HPLC fingerprint of YYTN.

**Figure 3:**
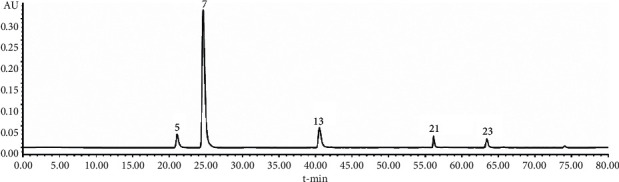
The HPLC fingerprint of mixed reference solution. Vanillic acid (*P5*), puerarin (*P7*), ferulic acid (*P13*), daidzein (*P21*), and formononetin (*P23*).

The HPLC fingerprints of the extracts of YYTN and their reference fingerprints are shown in Figure 1, and sample and mixed reference solution are shown in Figures 2 and 3, respectively. Twenty-four peaks with good separation and large peak areas were selected as common peaks to all the samples. The average retention time and peak areas of samples and the coefficient of variation (CV) of twenty-four peak areas are listed in Table 4. Values of CV were all more than 8.96%, which indicated that the content of each component of the samples under different extraction conditions varies greatly, especially the unknown compound represented by peak 2.

#### 3.1.3. Quantitative Analysis of YYTN

After comparing with the reference standard, five of the twenty-four common peaks were identified, which were vanillic acid (*P5*), puerarin (*P7*), ferulic acid (*P13*), daidzein (*P21*), and formononetin (*P23*). As shown in Table 5, quality control was performed on the contents of five compounds in nine samples.

#### 3.1.4. Similarity Analysis of Fingerprints

Each sample fingerprint of YYTN was analyzed by comparing with the reference fingerprint to evaluate the similarity among these samples. Results of similarity values between the reference and the nine samples are shown in Table 6.

### 3.2. Results of the Antioxidant Activity Test

#### 3.2.1. DPPH Assay

The IC_50_ values of DPPH radical scavenging activity test are listed in Table 7. The results revealed that nine extracts of YYTN solution exhibited the IC_50_ values that appeared within the range of 3.023–4.396 mg/mL. As shown in Table 7, S5 exhibited the most DPPH radical scavenging activity.

#### 3.2.2. FRAP Assay

According to the FRAP kit, the absorbance value of the FeSO_4_ standard was plotted on the abscissa, and the concentration corresponding to each absorbance value was plotted on the ordinate. The regression equation was as follows: *Y* = 3.4887*X* − 0.0291, *R*^2^ = 0.9994. The results revealed that the nine extracts of YYTN solution varied from 5.331 to 8.245 mmol/L equivalent to FeSO_4_. As shown in Table 7, S9 exhibited the highest ferric reducing antioxidant power.

### 3.3. Spectrum-Effect Relationship Results

#### 3.3.1. Pearson Product-Moment Correlation Coefficient

PPMCC analysis is performed with peak areas of twenty-four peaks as independent variables and IC_50_ values of DPPH radical scavenging percentage and FRAP values as dependent variables. Results of PPMCC are shown in Table 8. The PPMCC represents the correlation between the peak areas and the IC_50_ values of radical scavenging percentage and the FRAP values. If the PPMCC value was positive, it indicates that there was a positive correlation between independent and dependent variables. Conversely, if the PPMCC value is negative, then there is a negative correlation. The larger the absolute value of PPMCC, the better the correlation.

#### 3.3.2. Multiple Linear Regression Analysis

Using SPSS software, the peak areas of twenty-four common peaks were selected as independent variables and expressed as *P1*–*P24*, and IC_50_ values of DPPH radical scavenging percentage and FRAP values were selected as dependent variables and expressed as *Y1* and *Y2*, respectively. By stepwise regression analysis, the multiple regression equation of IC_50_ values of DPPH radical scavenging percentage variable was expressed as *Y1* = 0.308 − 0.004 × *P5* − 0.001 × *P6* + 0.001 × *P9* − 0.002 × *P13* *−* 0.004 × *P15* + 0.006 × *P16* − 0.001 × *P21* + 0.011 × *P24*, and the equation of FRAP was expressed as *Y2 ***=** −1.742 + 0.011 × *P5* + 0.008 × *P6−* × *P9* + 0.001 × *P13* − 0.026 × *P15* + 0.024 × *P16* − 0.001 × *P21* + 0.044 × *P24*. These two multivariate linear regression equations represent peaks that were strongly correlated with IC_50_ values of DPPH radical scavenging percentage and FRAP values. In the regression process, some peaks with no obvious correlation and multicollinearity were eliminated. Finally, the correlation peak was chosen to get the regression equation.

## 4. Discussion

YYTN is composed of *Radix Rehmanniae*, *Radix Astragali*, *Puerariae Lobatae Radix,* and *Chuanxiong Rhizoma*. *Radix Rehmanniae* had been found to have anticancer effects through promoting the activation of natural killer cells and dendritic cells [35, 36]. *Radix Astragali* has antitumor activity *in vitro* and *in vivo* [37]. It also exerts pharmacological activities on the cardiovascular, immune, respiratory, and hepatic systems [38]. *Puerariae Lobatae Radix* has been shown to be effective in the treatment of hypertension, cerebral ischemia, and diabetes [39–41]. *Chuanxiong Rhizoma* has a protective effect on diabetic nephropathy and has anticancer and antioxidant pharmacological activities [42, 43]. Therefore, YYTN, as the traditional Chinese herbal formula that combines these four Chinese medicines, has been extensively applied in the treatment of brain injury [3]. However, there are few reports on the HPLC fingerprint and pharmacological efficacy of YYTN.

From Figure 1 and Table 4, there were certain differences in the peak areas between the nine samples under different extraction conditions. However, as shown in Table 6, the similarity value of nine sample fingerprints and reference fingerprints ranged from 0.907 to 0.999, which indicated that the quality of the YYTN composed of *Radix Rehmanniae*, *Radix Astragali*, *Puerariae Lobatae Radix*, and *Chuanxiong Rhizoma* was relatively consistent and stable. We identified five compounds in YYTN by comparing Figures 2 and 3: *P5* was vanillic acid, *P7* was puerarin, *P13* was ferulic acid, *P21* was daidzein, and *P23* was formononetin. Subsequently, we carried out the quantitative determination of these compounds' content. The results are shown in Table 5: the contents of known components in YYTN were puerarin > formononetin > daidzein > vanillic acid > ferulic acid, indicating that puerarin was the most abundant in YYTN. The result of this experiment was consistent with that of previous research of our laboratory.

As shown in Table 7, DPPH radical scavenging percentage and FRAP values can reflect the antioxidant activity [44]. The results showed that YYTN had a good antioxidant activity. The literature showed that the antioxidant activity increased with the decrease of IC_50_ of DPPH radical scavenging percentage and the increase of FRAP values [45, 46]. According to the PPMCC shown in Table 8, *P4*, *P6*, *P7*, *P8*, *P9*, *P10*, *P12*, *P16*, *P21*, *P23*, and *P24* are positively correlated with DPPH scavenging percentage and FRAP values. *P6*, *P10*, and *P12* were negatively correlated with IC_50_ of DPPH radical scavenging percentage, while these peaks were positively correlated with FRAP. The order of correlation between peaks and IC_50_ of DPPH radical scavenging percentage followed *P12* > *P10* > *P6*, and FRAP values followed *P*6 > *P12* > *P10*. MLRA could eliminate the problem of multicollinearity that may exist among multiple independent variables. Therefore, MLRA analysis showed fewer peaks related to antioxidant activity than PPMCC. By MLRA, *P5*, *P6*, *P9*, *P13*, *P15*, *P16*, *P21*, and *P24* were related to IC_50_ of DPPH radical scavenging percentage and FRAP values. Among them, *P5*, *P6*, *P13*, *P15*, and *P24* were negatively correlated with IC_50_ of DPPH radical scavenging percentage, and *P5*, *P6*, *P13*, and *P24* were positively correlated with FRAP, which meant that *P5*, *P6*, *P13*, *P15*, and *P24* may have significant antioxidant activity. *P5* and *P13* were identified by HPLC as vanillic acid and ferulic acid, but *P6*, *P15*, and *P24* were not identified in this study. Therefore, we will further identify these three unknown components and prove whether these five peaks have a greater contribution to the antioxidant activity.

Because of the complexity of the components of traditional Chinese medicines, the chemical fingerprint cannot accurately evaluate the related information between the components and their efficacy [47, 48]. In this study, the fingerprints of YYTN were established, and no related reports were found in the literature. It was concluded that this study laid a foundation for the establishment of the fingerprint of YYTN. Subsequently, the antioxidant activity of YYTN was tested, which showed that YYTN had a certain extent of antioxidant activity and provided a certain therapeutic idea for the treatment of cardiovascular and cerebrovascular diseases because YYTN has been shown to be effective for cerebral ischemia and cardiac ischemia in animal experiments [49, 50]. Finally, the components of YYTN related to antioxidant activity were determined by correlation analysis, which may be significant to the research of the therapeutic material basis of YYTN.

Mathematical modeling is used in spectrum-effect relationship of traditional Chinese medicines [34]. For example, the two methods (PPMCC and MLRA) we used in this work indicated that both could establish the spectral correlation model to determine the chromatographic peaks which are closely related to the antioxidant activity. The two antioxidant methods mentioned in this work were also used in a lot of research [51–53]. The results showed that the methods were reliable for the determination of antioxidant activity *in vitro*. Of course, we will verify this result further in both cell and animal experiments *in vivo*. Our study not only established the spectrum-effect relationship, but also quantitative analysis of the active components in YYTN compared to other traditional Chinese medicine spectrum-effect correlations [20, 34].

In general, the main advantage of this research is that the relationship between HPLC fingerprints and the antioxidant activity of YYTN was established. Main antioxidant components of YYTN are further determined and would contribute to the quality standards of YYTN and to the study of the mechanism of other antioxidant components.

## 5. Conclusion

In the present study, we established the spectrum-effect relationship between the HPLC fingerprints and scavenging activity for DPPH and the total antioxidant activity for FRAP of YYTN. The HPLC analysis was used to build fingerprints of YYTN which contained up to twenty-four common peaks, and the similarity values of these fingerprints were evaluated by similarity analysis. The results showed that the similarity values of nine samples were more than 0.907, and puerarin was the most abundant chemical constituent in YYTN. The results of the spectrum-effect relationship indicate that peaks 5, 6, 13, 15, and 24 may be the main components responsible for the antioxidant activity of YYTN. This study shows that the fingerprinting method has been validated; it could provide a reliable and practical method for the consistency of quality of traditional Chinese medicine and other herbal preparations.

## Figures and Tables

**Table 1:** The herbal composition of YYTN simplified prescription.

Pharmaceutical name	Botanical name	Botanical family	Quantity (g)	Employed plant part
*Radix Rehmanniae*	*Rehmannia glutinosa Libosch.*	Scrophulariaceae	1	Tuberous root
*Radix Astragali*	*Astragalus membranaceus (Fisch.) Bge.*	Leguminosae	1	Root
*Puerariae Lobatae Radix*	*Pueraria lobata (Willd.) Ohwi*	Lamiaceae	1.2	Root
*Ligustici Chuanxiong Rhizoma*	*Ligusticum chuanxiong Hort.*	Apiaceae	0.67	Root and rhizome

**Table 2:** Independent variables and their levels.

Factors	Levels		
	1	2	3
Temperature (°C)	50	60	70
Extraction time (min)	40	50	60
Solvent-to-material ratio (mL/g)	6	7	8
Deviation	1	2	3

**Table 3:** The orthogonal table of sample solutions.

Run	Temperature (°C)	Extraction time (min)	Solvent-to-material ratio (mL/g)	Deviation
S1	1	1	1	1
S2	1	2	2	2
S3	1	3	3	3
S4	2	1	2	3
S5	2	2	3	1
S6	2	3	1	2
S7	3	1	3	2
S8	3	2	1	3
S9	3	3	2	1

**Table 4:** The peak area of each common peak to all the samples.

Peak no.	*t*_R_ (min)	Compounds	Mean peak area of each common peak (*n* = 3)										CV (%)
			*S*1	*S*2	*S*3	*S*4	*S*5	*S*6	*S*7	*S*8	*S*9	Control	
*P*1	4.35	Unknown	58.239	1598.968	1434.003	2242.623	1847.072	1928.97	1514.767	1870.101	1977.346	1608.01	37.13
*P*2	13.874	Unknown	894.038	124.294	106.241	153.788	127.161	149.786	112.926	141.261	149.642	217.682	110.10
*P*3	14.459	Unknown	158.758	485.685	410.196	602.872	477.105	570.061	429.368	537.156	520.098	465.7	26.48
*P*4	17.721	Unknown	2097.543	1797.045	1551.842	2169.958	1794.198	2087.783	1494.875	1850.784	1858.046	1622.726	12.73
*P*5	20.252	Vanillic acid	188.243	199.638	160.548	267.092	216.213	258.9	190.743	241.708	237.757	196.955	15.98
*P*6	23.059	Unknown	98.432	268.577	247.529	487.828	425.278	529.692	497.381	759.812	755.699	452.248	46.47
*P*7	23.891	Puerarin	12356.807	11137.137	9685.376	12278.256	10341.828	12285.794	9020.642	10978.242	10895.806	10997.765	10.16
*P*8	26.192	Unknown	3068.374	2724.584	2365.726	3034.727	2546.809	3021.683	2213.586	2713.51	2681.596	2472.933	10.92
*P*9	27.737	Unknown	2692.924	2497.148	2104.842	2746.072	2280.202	2812.948	2077.906	2645.69	2564.506	2491.36	10.39
*P*10	33.463	Unknown	451.595	431.389	415.448	893.588	757.391	812.497	767.16	924.589	988.359	715.78	29.60
*P*11	37.292	Unknown	55.412	208.42	138.712	237.401	195.599	230.8	189.65	238.356	233.033	191.932	29.67
*P*12	38.998	Unknown	118.244	134.402	218.107	382.948	320.283	391.466	352.623	444.97	479.304	315.816	39.00
*P*13	39.844	Ferulic acid	537.599	227.175	341.181	481.695	367.531	450.176	324.529	400.558	437.673	396.457	22.17
*P*14	44.968	Unknown	53.301	210.321	153.735	280.818	177.932	210.875	185.905	233.502	270.171	174.027	33.13
*P*15	46.175	Unknown	119.763	155.493	123.31	252.22	161.781	170.748	149.025	186.218	221.438	171.111	23.92
*P*16	46.762	Unknown	318.118	175.192	120.313	183.771	131.151	157.711	134.175	161.501	147.948	169.987	32.89
*P*17	47.891	Unknown	567.238	1400.02	1197.264	1678.034	1391.706	1645.648	1264.935	1542.522	1496.576	1290.745	23.53
*P*18	48.897	Unknown	102.963	171.029	160.95	196.841	163.064	193.266	142.509	174.395	174.906	152.996	16.49
*P*19	50.891	Unknown	541.894	225.149	194.53	275.116	230.743	267.933	190.102	258.261	244.444	209.586	38.67
*P*20	52.074	Unknown	480.788	483.885	403.028	540.935	453.536	531.823	424.461	547.367	501.954	431.888	10.64
*P*21	56.112	Daidzein	1632.799	617.779	593.706	561.935	520.579	618.128	268.638	354.741	442.295	623.4	60.14
*P*22	58.608	Unknown	232.051	245.562	204.692	333.709	276.466	323.545	212.518	301.423	304.541	244.717	17.31
*P*23	64.372	Formononetin	218.719	179.679	178.056	174.758	174.97	173.96	174.36	173.419	171.243	155.605	8.96
*P*24	68.493	Unknown	200.638	237.3	214.887	266.736	211.177	245.117	209.784	207.319	239.623	225.842	9.26

CV (%) = *σ*/*μ* × 100; *s* was the standard deviation; *μ* was the average value of the peak area. Control represents the control fingerprint automatically generated by the Similarity Evaluation System for Chromatographic Fingerprint of Traditional Chinese Medicine.

**Table 5:** The content of each group sample.

Compounds	Standard curve (*R*^2^)	The content of each group sample (mg/mL)								
		*S*1	*S*2	*S*3	*S*4	*S*5	*S*6	*S*7	*S*8	*S*9
Vanillic acid	*Y* = 1 × 10^7^*X* − 7.89 × 10^4^ (*R*^2^ = 0.999)	0.059	0.056	0.048	0.069	0.059	0.068	0.054	0.064	0.063
Puerarin	*Y* = 1 × 10^7^*X* − 1.30 × 10^4^ (*R*^2^ = 0.999)	2.397	2.230	1.940	2.458	2.071	2.460	1.807	2.198	2.182
Ferulic acid	*Y* = 3 × 10^7^*X* + 6.59 × 10^4^ (*R*^2^ = 0.998)	0.024	0.021	0.018	0.028	0.013	0.026	0.017	0.022	0.025
Daidzein	*Y* = 8 × 10^6^*X* + 9.69 × 10^4^ (*R*^2^ = 0.998)	0.124	0.130	0.124	0.116	0.106	0.130	0.043	0.064	0.086
Formononetin	*Y* = 2 × 10^6^*X* + 1.63 × 10^4^ (*R*^2^ = 0.999)	0.155	0.163	0.162	0.159	0.159	0.158	0.158	0.157	0.155

*R* ^2^ is the square of the correlation coefficient, which can judge the fitting degree of the linear regression line.

**Table 6:** Sample similarity analysis.

	*S*1	*S*2	*S*3	*S*4	*S*5	*S*6	*S*7	*S*8	*S*9	Control
*S*1	1	0.907	0.951	0.942	0.944	0.947	0.943	0.942	0.941	0.957
*S*2	0.907	1	0.956	0.954	0.954	0.955	0.953	0.952	0.952	0.964
*S*3	0.951	0.956	1	0.998	0.999	0.999	0.997	0.996	0.996	0.998
*S*4	0.942	0.954	0.998	1	1	0.999	0.999	0.998	0.998	0.998
*S*5	0.944	0.954	0.999	1	1	1	0.999	0.999	0.999	0.998
*S*6	0.947	0.955	0.999	0.999	1	1	0.999	0.999	0.998	0.999
*S*7	0.943	0.953	0.997	0.999	0.999	0.999	1	1	1	0.998
*S*8	0.942	0.952	0.996	0.998	0.999	0.999	1	1	1	0.997
*S*9	0.941	0.952	0.996	0.998	0.999	0.998	1	1	1	0.997
Control	0.957	0.964	0.998	0.998	0.998	0.999	0.998	0.997	0.997	1

This analysis was carried out with the software “Similarity Evaluation System for Chromatographic Fingerprint of Traditional Chinese Medicine.”

**Table 7:** The results of antioxidant activities ($\bar{x}$ ± SD, *n* = 3).

Sample no.	DPPH IC_50_ (mg/mL)	FRAP FeSO_4_ equivalent (mmol/L)
*S*1	3.775 ± 0.017	6.328 ± 0.025
*S*2	4.396 ± 0.024	6.170 ± 0.034
*S*3	3.290 ± 0.018	5.331 ± 0.029
*S*4	3.694 ± 0.011	7.242 ± 0.022
*S*5	3.023 ± 0.016	6.003 ± 0.019
*S*6	3.637 ± 0.016	7.762 ± 0.022
*S*7	3.307 ± 0.017	7.345 ± 0.025
*S*8	3.296 ± 0.022	7.959 ± 0.033
*S*9	3.116 ± 0.017	8.245 ± 0.032

**Table 8:** PPMCC between the twenty-four common peaks of YYTN and antioxidant activities.

Factor	Peak number																							
	*P1*	*P2*	*P3*	*P4*	*P5*	*P6*	*P7*	*P8*	*P9*	*P10*	*P11*	*P12*	*P13*	*P14*	*P15*	*P16*	*P17*	*P18*	*P19*	*P20*	*P21*	*P22*	*P23*	*P24*
*DPPH*																								
IC_50_ (mg/mL)	−0.2435	0.2430	−0.1080	0.3392	−0.0309	−0.4989	0.4827	0.4651	0.3832	−0.5174	−0.1174	−0.6136	−0.2102	−0.1070	−0.1094	0.4606	−0.1174	−0.0125	0.2710	0.2299	0.3774	−0.0647	0.3425	0.3301
*FRAP*																								
FeSO_4_ (mmol/L)	−0.2473	−0.1771	−0.4057	−0.5325	−0.2092	0.5277	−0.6005	−0.5876	−0.3878	0.3466	0.0211	0.3863	−0.1669	−0.0786	−0.1410	−0.4685	−0.3621	−0.6388	−0.3650	−0.2388	−0.7714	−0.2389	−0.5031	−0.6329

## Data Availability

The data used to support the findings of this study are available from the corresponding author upon request.
